# Psychotropic Medication Prescribing for Children and Adolescents After the Onset of the COVID-19 Pandemic

**DOI:** 10.1001/jamanetworkopen.2024.7965

**Published:** 2024-04-23

**Authors:** Zaba Valtuille, Eric Acquaviva, Vincent Trebossen, Naim Ouldali, Aurelie Bourmaud, Stéphane Sclison, Alexandre Gomez, Alexis Revet, Hugo Peyre, Richard Delorme, Florentia Kaguelidou

**Affiliations:** 1 Center of Clinical Investigations, Inserm CIC1426, Robert Debré University Hospital, APHP.Nord, Paris, France; 2 Paris Cité University, EA7323 Perinatal and Pediatric Pharmacology and Therapeutic Assessment, Paris, France; 3 Department of Child and Adolescent Psychiatry, Robert Debré University Hospital, APHP.Nord, Paris Cité University, Paris, France; 4 Department of General Pediatrics, Pediatric Infectious Disease and Internal Medicine, Robert Debré University Hospital, APHP.Nord, Paris Cité University, Paris, France; 5 Clinical Epidemiology Unit, Inserm CIC1426, Robert Debré University Hospital, APHP.Nord, Paris Cité University, Paris, France; 6 Consulting Services & Analytics Department, IQVIA, Courbevoie, France; 7 Department of Child and Adolescent Psychiatry, Toulouse University Hospital, Toulouse, France; 8 CERPOP, UMR 1295, Inserm, Toulouse III - Paul Sabatier University, Toulouse, France; 9 Autism Reference Centre of Languedoc-Roussillon CRA-LR, Montpellier University Hospital, Montpellier, France; 10 Excellence Centre for Autism and Neurodevelopmental disorders- CeAND, MUSE University, Montpellier, France; 11 Université Paris-Saclay, UVSQ, Inserm, CESP, Team DevPsy, Villejuif, France; 12 Human Genetics & Cognitive Functions, Institut Pasteur, Paris, France

## Abstract

**Importance:**

Numerous studies have provided evidence for the negative associations of the COVID-19 pandemic with mental health, but data on the use of psychotropic medication in children and adolescents after the onset of the COVID-19 pandemic are lacking.

**Objective:**

To assess the rates and trends of psychotropic medication prescribing before and over the 2 years after the onset of the COVID-19 pandemic in children and adolescents in France.

**Design, Setting, and Participants:**

This cross-sectional study used nationwide interrupted time-series analysis of outpatient drug dispensing data from the IQVIA X-ponent database. All 8 839 143 psychotropic medication prescriptions dispensed to children (6 to 11 years of age) and adolescents (12 to 17 years of age) between January 2016 and May 2022 in France were retrieved and analyzed.

**Exposure:**

Onset of COVID-19 pandemic.

**Main outcomes and Measures:**

Monthly rates of psychotropic medication prescriptions per 1000 children and adolescents were analyzed using a quasi-Poisson regression before and after the pandemic onset (March 2020), and percentage changes in rates and trends were assessed. After the pandemic onset, rate ratios (RRs) were calculated between estimated and expected monthly prescription rates. Analyses were stratified by psychotropic medication class (antipsychotic, anxiolytic, hypnotic and sedative, antidepressant, and psychostimulant) and age group (children, adolescents).

**Results:**

In total, 8 839 143 psychotropic medication prescriptions were analyzed, 5 884 819 [66.6%] for adolescents and 2 954 324 [33.4%] for children. In January 2016, the estimated rate of monthly psychotropic medication prescriptions was 9.9 per 1000 children and adolescents, with the prepandemic rate increasing by 0.4% per month (95% CI, 0.3%-0.4%). In March 2020, the monthly prescription rate dropped by 11.5% (95% CI, −17.7% to −4.9%). During the 2 years following the pandemic onset, the trend changed significantly, and the prescription rate increased by 1.3% per month (95% CI, 1.2%-1.5%), reaching 16.1 per 1000 children and adolescents in May 2022. Monthly rates of psychotropic medication prescriptions exceeded the expected rates by 11% (RR, 1.11 [95% CI, 1.08-1.14]). Increases in prescribing trends were observed for all psychotropic medication classes after the pandemic onset but were substantial for anxiolytics, hypnotics and sedatives, and antidepressants. Prescription rates rose above those expected for all psychotropic medication classes except psychostimulants (RR, 1.12 [95% CI, 1.09-1.15] in adolescents and 1.06 [95% CI, 1.05-1.07] in children for antipsychotics; RR, 1.30 [95% CI, 1.25-1.35] in adolescents and 1.11 [95% CI, 1.09-1.12] in children for anxiolytics; RR, 2.50 [95% CI, 2.23-2.77] in adolescents and 1.40 [95% CI, 1.30-1.50] in children for hypnotics and sedatives; RR, 1.38 [95% CI, 1.29-1.47] in adolescents and 1.23 [95% CI, 1.20-1.25] in children for antidepressants; and RR, 0.97 [95% CI, 0.95-0.98] in adolescents and 1.02 [95% CI, 1.00-1.04] in children for psychostimulants). Changes were more pronounced among adolescents than children.

**Conclusions and Relevance:**

These findings suggest that prescribing of psychotropic medications for children and adolescents in France significantly and persistently increased after the COVID-19 pandemic onset. Future research should identify underlying determinants to improve psychological trajectories in young people.

## Key Points

**Question:**

How did prescribing of psychotropic medications for children and adolescents change in the 2 years following the onset of the COVID-19 pandemic?

**Findings:**

This cross-sectional study retrieved and analyzed all 8 839 143 psychotropic medication prescriptions dispensed to individuals aged from 6 to 17 years in France between 2016 and 2022. An interrupted time-series analysis showed steady increases in prescription trends for all psychotropic medications after the pandemic onset, with prescription rates of all psychotropic medication classes except psychostimulants higher than expected rates.

**Meaning:**

Findings of significant increases in prescribing of psychotropic medications for children and adolescents after the pandemic onset underscore the need for future research to identify the underlying determinants.

## Teaser

This cross-sectional study assesses whether the rates and trends of psychotropic medication prescribing changed from before vs in the 2 years after the onset of the COVID-19 pandemic for children and adolescents.

## Introduction

Mental health disorders are identified as one of the most significant challenges in pediatric health care.^1,2^ Their prevalence has been increasing over the years, and currently almost 15% of children and adolescents are diagnosed with a mental disorder by the age of 18 years.^3^ In this context, the COVID-19 pandemic has been a major aggravating factor with an unprecedented, large-scale negative impact on mental health. Several studies have described a substantial deterioration of mental well-being in children and young people after the onset of the pandemic, with increases in health care needs.^4,5,6^

Use of psychotropic medications is often considered a proxy for the mental health status of a population or for the prevalence of specific mental disorders.^7^ Population-based studies have reported increases in the uptake of antidepressants and hypnotics among youths (<18 or 19 years) in the months following the pandemic onset.^8,9,10^ Increases in overall psychotropic medication consumption, more important in the youngest age groups, were estimated at 35% among children 10 to 14 years of age and 15 to 19 years.^11,12,13^ However, data on psychotropic medication use in children and adolescents long after the pandemic onset are currently lacking. The purpose of the present study was to assess the rates and trends of psychotropic medication prescribing for children and adolescents before and in the 2 years after the onset of the COVID-19 pandemic in France.

## Methods

### Study Design and Setting

This cross-sectional study used an interrupted time-series analysis of outpatient drug dispensing data from January 1, 2016, to May 31, 2022. This study was part of the MENTALPED national project,^14^ financed in 2021, to evaluate the association between the COVID-19 pandemic and the use of pediatric mental health medical resources by children and adolescents. The project’s protocol was approved by the national Ethics and Scientific Committee for Health Research, Studies and Evaluations and by the French Data Protection Authority and is publicly available in the ClinicalTrials.gov database.^14^ The requirement to obtain patient informed consent was waived because the study used deidentified data and no patients were contacted. Findings were reported following the Strengthening the Reporting of Observational Studies in Epidemiology (STROBE) reporting guideline.^15^

### Study Data Sources and Population

Study data originated from the X-ponent database (IQVIA; France), a privately owned database that contains information on prescriptions dispensed from a sample of 14 000 pharmacies, approximately 60% of all retail pharmacies in France, excluding overseas territories.^16^ Pharmacies are selected through cluster sampling stratified by region and activity volume to ensure representativeness on a national level. Data are extrapolated through an internal projection algorithm to approximate national estimates of drug prescription fills as detailed elsewhere.^17^

We extracted all psychotropic medication prescriptions dispensed to pediatric outpatients aged from 6 to 17 years during the study period. Children were defined as individuals aged 6 through 11 years; adolescents, 12 through 17 years. Dispensed prescriptions were identified as all pharmacy sales records containing at least 1 psychotropic medication based on the Anatomical Therapeutic Chemical classification: antipsychotics (N05A), anxiolytics (N05B), hypnotics and sedatives (N05C except N05CH01, melatonin), antidepressants (N06A), and psychostimulants (N06B) (eTable 1 in Supplement 1). For each dispensed prescription, we retrieved the dispensed medication (Anatomical Therapeutic Chemical fifth level) and the patient’s age at dispensing.

### Outcome Measures and Study Periods

The monthly rates of psychotropic medication prescriptions per 1000 children and adolescents in France were calculated using the counts of dispensed prescriptions (numerator) and appropriate age-specific population estimates derived from the annual dataset of the National Institute of Statistics and Economic Studies (denominator).^18^ Both the numerator and denominator were aggregated on a monthly level.

The study involved 3 predefined periods: the period before the pandemic (prepandemic) from January 1, 2016, to February 29, 2020, and 2 pandemic periods: the initial pandemic period from March 1 to May 31, 2020 (corresponding to the strict home confinement of the population and responsible for major disruptions in health care), and the period after the pandemic onset from June 1, 2020, to May 31, 2022.

### Statistical Analysis

We assessed changes in the monthly rate (level) and trend (slope) of psychotropic medication prescriptions before and after the pandemic onset using a quasi-Poisson regression accounting for autocorrelation and prepandemic trends.^19,20^ Seasonal variations in psychotropic medication dispensing may be due to seasonal fluctuations of clinical symptoms, prescribing patterns, and practical considerations. Therefore, seasonality was addressed by fitting calendar months as a categorical variable. Residual autocorrelation in the errors was assessed through visual inspection of correlograms (autocorrelation and partial autocorrelation functions plots) and was corrected, when present, by using the Newey-West method.^21^

We hypothesized that there would be an immediate change in the prescription rate (level change) at the beginning of every pandemic period and a progressive change (slope change) in the period after the pandemic onset. The interrupted time-series model, therefore, included time as a continuous variable to assess the baseline trend, 2 binary variables indicating the 2 pandemic periods to measure the level changes, and an interaction term between time and the postpandemic binary variable to measure the slope change. The validity of all regression models was assessed by visual inspection of correlograms and appropriate residual analysis. Model parameters were interpreted as percentage changes with 95% CIs. A linear combination of the trend parameters (prepandemic slope and estimated slope change) was implemented to obtain the trend after the onset of the pandemic with 95% CIs.^20^ The monthly prescription rates estimated by the model during the 2-year period after the onset of the pandemic were also compared with the expected rates forecast by the model based on prepandemic data only. Results were expressed as rate ratios (RRs) with corresponding 95% CIs calculated by bootstrapping (n = 10 000 replications) (eMethods in Supplement 1). Analyses were further stratified by psychotropic medication class (antipsychotics, anxiolytics, hypnotics and sedatives, antidepressants, and psychostimulants) and age group (6-11 years and 12-17 years).

Finally, we performed 2 sensitivity analyses to evaluate the robustness of our results. First, we accounted for seasonality in the quasi-Poisson model by applying Fourier terms, and second, we repeated the analysis using a negative binomial regression accounting for seasonality and autocorrelation.

All statistical tests were 2-sided, and *P* < .05 was considered statistically significant. All analyses were performed using R software, version 4.2.2 (R Project for Statistical Computing).

## Results

Between January 1, 2016, and May 31, 2022, 8 839 143 psychotropic medication prescriptions were dispensed: 2 468 197 (27.9%) antipsychotics, 1 971 822 (22.3%) anxiolytics, 81 387 (0.9%) hypnotics and sedatives, 1 342 041 (15.2%) antidepressants, and 3 803 770 (43.0%) psychostimulants. The majority of these prescriptions were dispensed to adolescents (5 884 819 [66.6%]; 2 954 324 prescriptions [33.4%] to children).

### Changes in Overall Psychotropic Medication Prescription Rates

**Figure 1.**
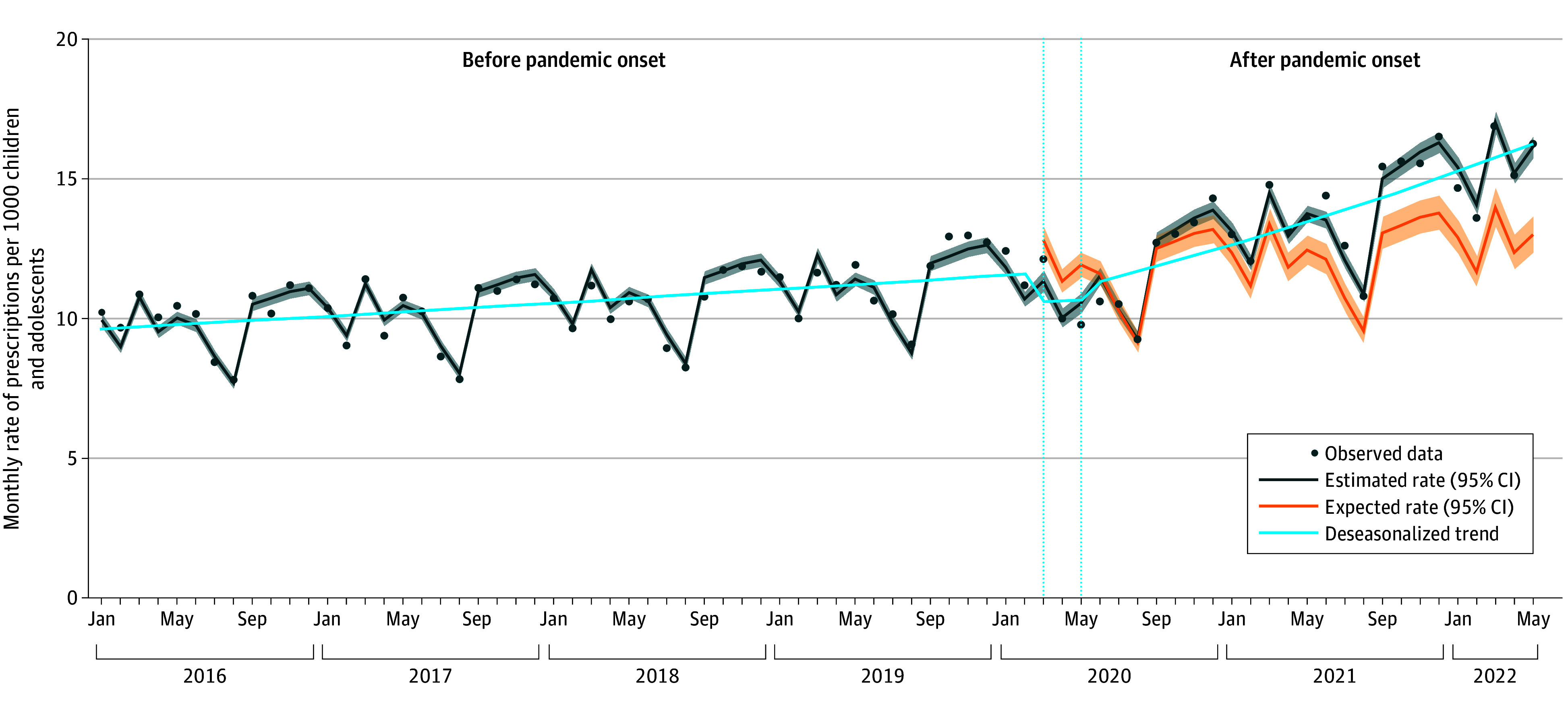
Changes in Rates and Trends of Monthly Psychotropic Medication Prescriptions for Children and Adolescents in France Estimated monthly prescription rates (dark gray line) were based on observed data using the quasi-Poisson regression model with 95% CIs. Monthly expected rates (orange line) were based on prepandemic observed data using the quasi-Poisson model with 95% CIs. Prescription trends (blue line) were estimated using a deseasonalized quasi-Poisson regression model. The initial pandemic period (vertical dotted lines) was between March and May 2020.

In January 2016, the estimated rate of monthly psychotropic medication prescriptions was 9.9 per 1000 children and adolescents in France, and the prepandemic monthly prescription rate was increasing by 0.4% per month (95% CI, 0.3%-0.4%) (Table, Figure 1). In the initial pandemic period, the monthly rate of psychotropic medication prescriptions immediately dropped by 11.5% (95% CI, −17.7% to −4.9%). After the pandemic onset, the trend significantly changed, and the rate increased by 1.3% per month (95% CI, 1.2%-1.5%), reaching 16.1 psychotropic medication prescriptions per 1000 children and adolescents in May 2022. Monthly rates of psychotropic medication prescriptions after the pandemic onset exceeded the expected rates by 11% (RR, 1.11 [95% CI, 1.08-1.14]).

**Table.** Changes in Rates and Trends of Psychotropic Medication Prescriptions

Outcome	Before the pandemic onset (January 2016 through February 2020)			Initial pandemic period (March through May 2020)		After the pandemic onset (June 2020 through May 2022)					
	Level January 2016 (95% CI)^a^	Trend, % (95% CI)^b^	*P* value	Change in level, % (95% CI)^b^	*P* value	Change in level, % (95% CI)^b^	*P* value	Change in trend, % (95% CI)^b^	*P* value	Trend, % (95% CI)^b^	Level May 2022 (95% CI)^a^
Monthly prescription rate of psychotropic medications	9.9 (9.6 to 10.2)	0.4 (0.3 to 0.4)	<.001	−11.5 (−17.7 to −4.9)	<.001	−1.7 (−5.1 to 1.8)	.34	1.0 (0.8 to 1.2)	<.001	1.3 (1.2 to 1.5)	16.1 (15.7 to 16.6)
Children	6.2 (6.0 to 6.4)	0.5 (0.4 to 0.5)	<.001	−11.7 (−16.1 to −7.1)	<.001	−3.0 (−6.5 to 0.7)	.12	0.6 (0.4 to 0.9)	<.001	1.1 (0.9 to 1.3)	10.5 (10.2 to 10.8)
Adolescents	13.6 (13.2 to 14.1)	0.3 (0.2 to 0.4)	<.001	−11.5 (−18.1 to −4.4)	.002	−1.2 (−4.8 to 2.5)	.51	1.1 (0.9 to 1.3)	<.001	1.4 (1.3 to 1.6)	21.6 (21.0 to 22.3)
Monthly prescription rate of antipsychotics	2.6 (2.5 to 2.7)	0.3 (0.2 to 0.4)	<.001	−1.6 (−9.0 to 6.4)	.69	1.1 (−2.2 to 4.5)	.52	0.7 (0.6 to 0.9)	<.001	1.0 (0.9 to 1.2)	4.1 (4.0 to 4.3)
Children	1.2 (1.2 to 1.2)	0.4 (0.3 to 0.5)	<.001	−2.0 (−9.9 to 6.5)	.63	1.4 (−1.5 to 4.4)	.36	0.3 (0.2 to 0.5)	<.001	0.7 (0.6 to 0.9)	1.9 (1.9 to 2.0)
Adolescents	4.0 (3.9 to 4.1)	0.3 (0.2 to 0.4)	<.001	−1.6 (−8.9 to 6.2)	.67	0.7 (−2.7 to 4.3)	.69	0.8 (0.7 to 1.0)	<.001	1.1 (1.0 to 1.3)	6.3 (6.1 to 6.5)
Monthly prescription rate of anxiolytics	2.7 (2.6 to 2.8)	−0.2 (−0.3 to −0.1)	<.001	−13.6 (−17.7 to −9.3)	<.001	8.4 (3.9 to 13.0)	<.001	1.1 (0.9 to 1.4)	<.001	0.9 (0.7 to 1.1)	3.2 (3.1 to 3.3)
Children	1.5 (1.5 to 1.5)	−0.1 (−0.2 to 0.0)	.03	−12.2 (−15.5 to −8.7)	<.001	5.1 (1.2 to 9.2)	.01	0.4 (0.2 to 0.6)	<.001	0.3 (0.1 to 0.4)	1.5 (1.4 to 1.5)
Adolescents	3.9 (3.8 to 4.1)	−0.2 (−0.3 to 0.1)	<.001	−14.4 (−19.6 to −8.9)	<.001	9.3 (4.4 to 14.6)	<.001	1.4 (1.1 to 1.6)	<.001	1.1 (0.9 to 1.4)	4.8 (4.7 to 5.0)
Monthly prescription rate of hypnotics and sedatives	0.2 (0.2 to 0.2)	−2.8 (−3.1 to −2.5)	<.001	40.9 (22.8 to 61.6)	<.001	43.0 (24.9 to 63.7)	<.001	3.9 (3.4 to 4.4)	<.001	1.0 (0.7 to 1.4)	0.1 (0.1 to 0.1)
Children	0.0 (0.0 to 0.0)	−1.5 (−1.8 to 1.2)	<.001	12.1 (−12.1 to 41.5)	.35	−0.2 (−17.0 to 19.6)	.98	2.6 (1.5 to 3.8)	<.001	1.0 (0.0 to 2.1)	0.0 (0.0 to 0.0)
Adolescents	0.4 (0.3 to 0.4)	−2.9 (−3.2 to −2.6)	<.001	43.6 (24.1 to 66.2)	<.001	47.7 (27.8 to 70.7)	<.001	4.0 (3.5 to 4.5)	<.001	1.0 (0.6 to 1.3)	0.2 (0.1 to 0.2)
Monthly prescription rate of antidepressants	1.1 (1.1 to 1.2)	0.5 (0.4 to 0.7)	<.001	−1.8 (−9.8 to 6.9)	.67	3.3 (−2.0 to 9.0)	.23	2.2 (2.0 to 2.5)	<.001	2.8 (2.6 to 3.0)	3.3 (3.2 to 3.4)
Children	0.3 (0.3 to 0.3)	0.1 (−0.1 to 0.2)	.42	2.8 (−2.4 to 8.3)	.30	12.0 (6.2 to 18.0)	<.001	0.7 (0.4 to 1.0)	<.001	0.8 (0.5 to 1.1)	0.4 (0.4 to 0.4)
Adolescents	2.0 (1.9 to 2.1)	0.6 (0.4 to 0.7)	<.001	−2.6 (−10.8 to 6.4)	.56	2.2 (−3.1 to 7.8)	.42	2.3 (2.1 to 2.6)	<.001	2.9 (2.7 to 3.1)	6.1 (5.9 to 6.3)
Monthly prescription rate of psychostimulants	4.1 (3.9 to 4.2)	0.7 (0.6 to 0.8)	<.001	−18.4 (−23.0 to −13.7)	<.001	−8.5 (−12.3 to −4.6)	<.001	0.6 (0.4 to 0.9)	<.001	1.4 (1.1 to 1.6)	7.3 (7.1 to 7.6)
Children	3.5 (3.4 to 3.6)	0.7 (0.6 to 0.8)	<.001	−14.9 (−19.7 to −9.9)	<.001	−6.6 (−10.4 to −2.6)	.002	0.7 (0.4 to 0.9)	<.001	1.4 (1.2 to 1.7)	7.1 (6.9 to 7.4)
Adolescents	4.6 (4.5 to 4.8)	0.7 (0.6 to 0.8)	<.001	−21.5 (−26.0 to −16.7)	<.001	−10.4 (−14.3 to −6.3)	<.001	0.6 (0.3 to 0.9)	<.001	1.3 (1.1 to 1.6)	7.5 (7.3 to 7.8)

^a^ Level corresponds to the monthly prescription rate per 1000 children and adolescents estimated by the model.

^b^ Model estimates for trend and changes in level and trend are presented as percentage changes per month (95% CI).

**Figure 2.**
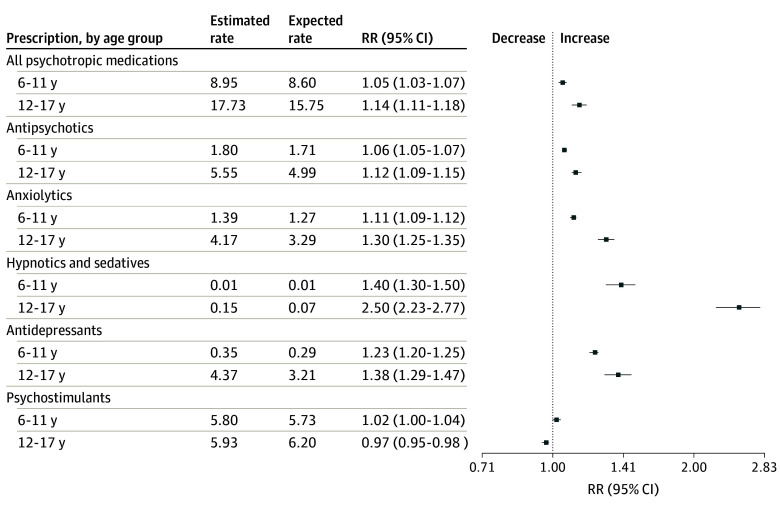
Rate Ratios (RRs) and 95% CIs of Estimated and Expected Monthly Rates of Psychotropic Medication Prescriptions for Children and Adolescents in the 2 Years Following Pandemic Onset Estimated monthly prescription rates were fitted by the model based on observed data, and expected monthly rates were forecast by the model based only on prepandemic observed data. Vertical dotted line indicates no relative change between estimated and expected rates (RR = 1).

Compared with changes in children, changes were more pronounced in adolescents, with prescription trends increasing from 0.3% per month (95% CI, 0.2%-0.4%) in the prepandemic period to 1.4% per month (95% CI, 1.3%-1.6%) in the period after the pandemic onset (Table; eFigure 1 in Supplement 1). Compared with the expected rates, monthly prescription rates presented a relative increase of 14% (RR, 1.14 [95% CI, 1.11-1.18]) in adolescents and 5% (RR, 1.05 [95% CI, 1.03-1.07]) in children (Figure 2). Details of the estimates of the time-series model overall and by age group and specific psychotropic medication class are provided in eTable 2 in Supplement 1.

### Changes in the Prescription Rates of Antipsychotics

**Figure 3.**
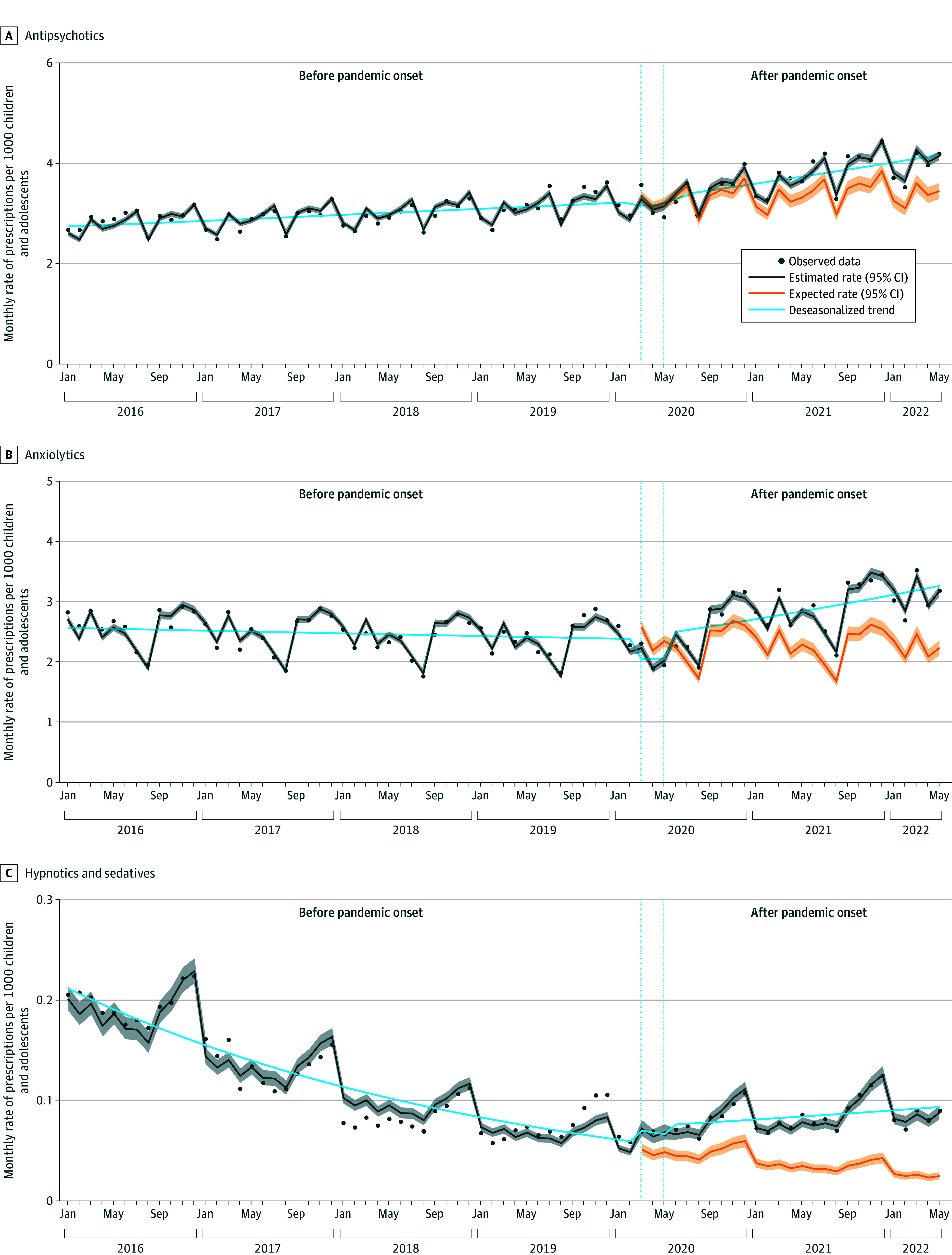
Changes in Rates and Trends of Monthly Psycholeptic Medication Prescriptions for Children and Adolescents in France Prescription rates (dark gray line) based on observed data using quasi-Poisson regression with 95% CIs. Expected rates (orange line) based on prepandemic observed data using quasi-Poisson model with 95% CIs. Prescription trends (blue line) estimated using deseasonalized quasi-Poisson regression.Vertical dotted lines indicate initial pandemic period.

In January 2016, the estimated rate of antipsychotic prescriptions was 2.6 per 1000 children and adolescents, and the prepandemic rate was increasing by 0.3% per month (95% CI, 0.2%-0.4%) (Table, Figure 3). The monthly rate of prescriptions did not significantly change in the initial pandemic period nor after the pandemic onset. However, the trend significantly changed, and the antipsychotic medication prescription rate increased by 1.0% per month (95% CI, 0.9%-1.2%), reaching 4.1 per 1000 children and adolescents in May 2022. Compared with the expected rates, a relative increase of 11% (RR, 1.11 [95% CI, 1.09-1.13]) was observed.

After the pandemic onset, the trend change was more pronounced in adolescents than in children (Table; eFigure 2 in Supplement 1). In addition, monthly rates of prescriptions exceeded the expected rates by 12% (RR, 1.12 [95% CI, 1.09-1.15]) in adolescents and 6% (RR, 1.06 [95% CI, 1.05-1.07]) in children (Figure 2).

### Changes in the Prescription Rates of Anxiolytics

In January 2016, the estimated rate of anxiolytic prescriptions was 2.7 per 1000 children and adolescents, and the prepandemic rate was decreasing by 0.2% per month (95% CI, −0.3% to −0.1%) (Table, Figure 3). After an immediate drop in the monthly prescription rate during the initial pandemic period of 13.6% (95% CI, −17.7% to −9.3%), the trend substantially changed after the pandemic onset, and the anxiolytic prescription rate increased by 0.9% per month (95% CI, 0.7%-1.1%), reaching 3.2 per 1000 children and adolescents in May 2022. Compared with the expected rates, a relative increase of 25% (RR, 1.25 [95% CI, 1.21-1.29]) was observed.

Similar patterns of level and trend changes were observed in children and adolescents, although estimates were higher in adolescents than in children both before and after the pandemic onset (Table; eFigure 3 in Supplement 1). Monthly rates of anxiolytic prescriptions exceeded the expected rates by 30% (RR, 1.30 [95% CI, 1.25-1.35]) in adolescents and 11% (RR, 1.11 [95% CI, 1.09-1.12]) in children (Figure 2).

### Changes in the Prescription Rates of Hypnotics and Sedatives

In January 2016, the estimated rate of hypnotic and sedative prescriptions was 0.2 per 1000 children and adolescents, and the prepandemic rate was decreasing by 2.8% per month (95% CI, −3.1% to −2.5%) (Table, Figure 3). The monthly rate immediately increased during the initial pandemic period by 40.9% (95% CI, 22.8%-61.6%). Thereafter, the trend changed significantly, and the prescription rate increased by 1.0% per month (95% CI, 0.7%-1.4%), reaching 0.1 per 1000 children and adolescents in May 2022. Monthly rates of prescriptions exceeded the expected rates by 138% (RR, 2.38 [95% CI, 2.14-2.63]).

The change in trend after the pandemic onset was more pronounced in adolescents than in children, and rate estimates were also higher in adolescents (Table; eFigure 4 in Supplement 1). Monthly rates of hypnotic and sedative prescriptions exceeded the expected rates by 150% (RR, 2.50 [95% CI, 2.23-2.77]) in adolescents and by 40% (RR, 1.40 [95% CI, 1.30-1.50]) in children (Figure 2).

### Changes in the Prescription Rates of Antidepressants

**Figure 4.**
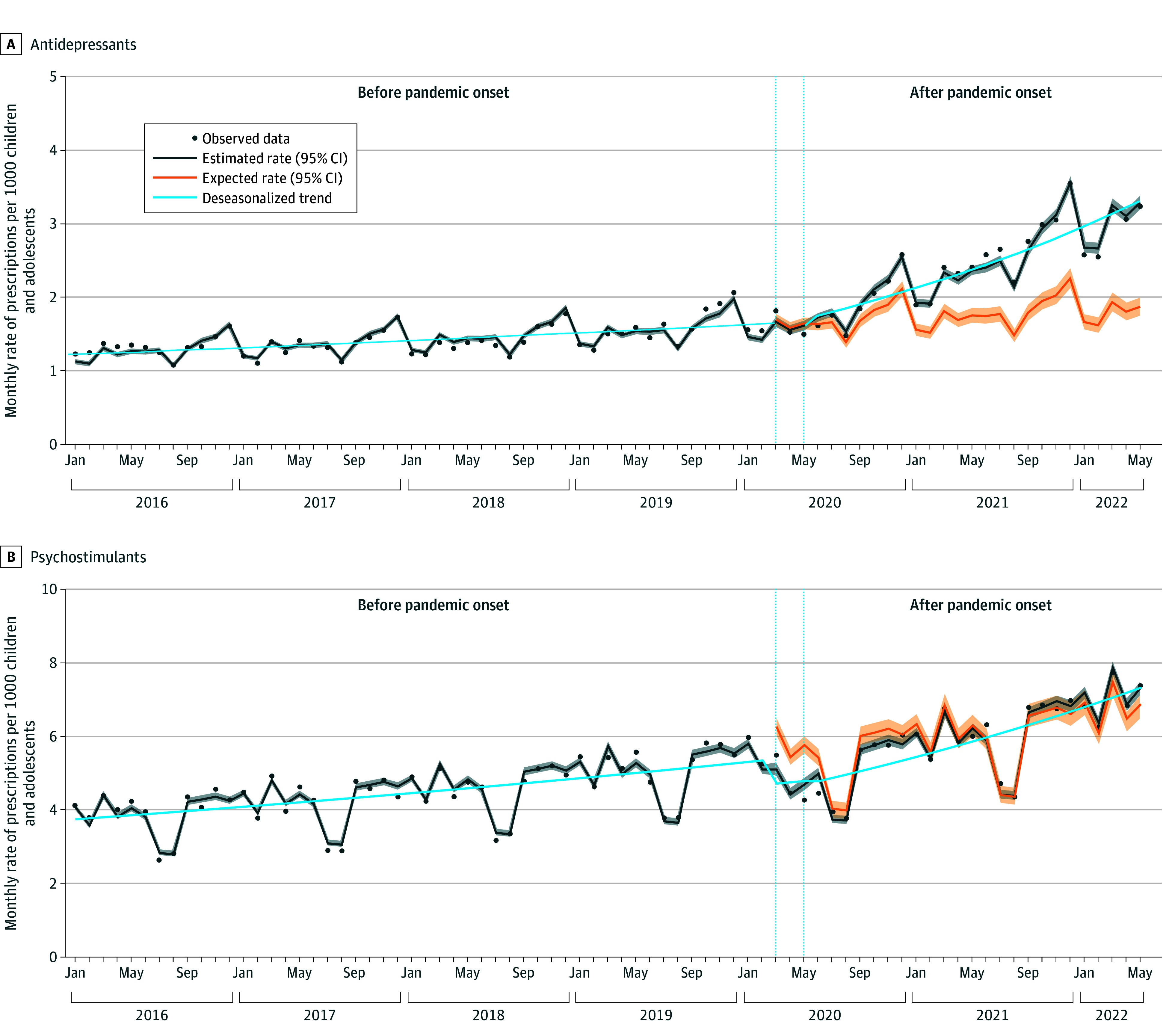
Changes in Rates and Trends of Monthly Psychoanaleptic Medication Prescriptions for Children and Adolescents in France Estimated monthly prescription rates (dark gray line) were based on observed data using the quasi-Poisson regression model with 95% CIs. Monthly expected rates (orange line) were based on prepandemic observed data using the quasi-Poisson model with 95% CIs. Prescription trends (blue line) were estimated using a deseasonalized quasi-Poisson regression model. The initial pandemic period (vertical dotted lines) was between March and May 2020.

In January 2016, the estimated rate of antidepressant prescriptions was 1.1 per 1000 children and adolescents, and the prepandemic rate was increasing by 0.5% per month (95% CI, 0.4%-0.7%) (Table, Figure 4). The monthly rate of prescriptions did not significantly change in the initial pandemic period nor after the pandemic onset. However, the trend significantly changed, and the prescription rate increased by 2.8% per month (95% CI, 2.6%-3.0%), reaching 3.3 per 1000 children and adolescents in May 2022. Monthly rates of prescriptions exceeded the expected rates by 38% (RR, 1.38 [95% CI, 1.30-1.47]).

After the pandemic onset, the trend change was more pronounced in adolescents than in children, and rate estimates were also substantially higher in adolescents (Table; eFigure 5 in Supplement 1). Monthly rates of antidepressant prescriptions exceeded the expected rates by 38% (RR, 1.38 [95% CI, 1.29-1.47]) in adolescents and 23% (RR, 1.23 [95% CI, 1.20-1.25]) in children (Figure 2).

### Changes in the Prescription Rates of Psychostimulants

In January 2016, the estimated rate of psychostimulant prescriptions was 4.1 per 1000 children and adolescents, and the prepandemic rate was increasing by 0.7% per month (95% CI, 0.6%-0.8%) (Table, Figure 4). In the initial pandemic period, the monthly prescription rate immediately dropped by 18.4% (95% CI, −23.0% to −13.7%). Thereafter, the trend significantly changed, and the prescription rate increased by 1.4% per month (95% CI, 1.1%-1.6%), reaching 7.3 prescriptions per 1000 children and adolescents in May 2022. Compared with the expected rates, monthly prescription rates did not significantly change (RR, 0.99 [95% CI, 0.97-1.01]).

Children and adolescents displayed similar patterns and estimates of level and trend changes for psychostimulant prescription rates (Table; eFigure 6 in Supplement 1). However, compared with the expected rates, psychostimulant prescription rates decreased by 3% (RR, 0.97 [95% CI, 0.95-0.98]) in adolescents and did not significantly change in children (RR, 1.02 [95% CI, 1.00-1.04]) (Figure 2).

### Sensitivity Analyses

We conducted 2 sensitivity analyses to evaluate the robustness of our results. Both sensitivity analyses yielded model estimates for level and trend changes during the study period that were consistent with those of the primary analysis (eTable 3 and eFigure 7 in Supplement 1).

## Discussion

This cross-sectional study found that, overall, psychotropic medication prescribing for children and adolescents in France significantly and persistently increased after the onset of the COVID-19 pandemic. Increases in prescription trends were substantial for anxiolytics, hypnotics and sedatives, and antidepressants and moderate for antipsychotics and psychostimulants. Prescription rates rose above the expected rates for all psychotropic medication classes except psychostimulants. Changes were more pronounced in adolescents than in children.

The COVID-19 pandemic had a well-documented negative impact on the mental health status of children and adolescents.^22,23^ Numerous studies worldwide have reported increases mainly in the prevalence of anxiety and depressive symptoms in young people^24,25^ and in the number of hospitalizations and emergency department visits for severe mood disorders and suicidality in the year following the pandemic onset.^10,26,27,28,29^ Consequent increments in psychotropic medication prescribing have been observed, often after a transient decrease during strict lockdowns,^11,12,13,30,31^ but few studies have data to report on persisting effects associated with the pandemic. A Danish study^32^ reported increases in the incident use of psychotropic medications (18%), mainly hypnotics, antidepressants, and psychostimulants, among children and adolescents up to mid-2022. In Finland, increases have been reported, but without precise estimations, in the use of antidepressant and psychostimulant medications among children aged 6 to 12 years up to March 2022.^33^

Our findings are consistent with a rise in psychotropic medication prescribing for children and adolescents during the 2 years after the pandemic onset compared with prepandemic rates and trends. The most substantial increases in rates and trends of prescriptions concerned anxiolytic, hypnotic and sedative, and antidepressant medications. This finding is in line with the growing prevalence of mood and anxiety disorders recently reported in the pediatric population, whose first-line treatment often involves the prescribing of these specific psychotropic medication classes.^8,30,34,35,36^ Before the pandemic onset, anxiolytics and hypnotics and sedatives were the only psychotropic medication classes with decreasing prescription trends in the pediatric population in France. This decrease probably accounts for the substantial relative increases observed after the pandemic onset even though prescription rates remained low overall, especially those of hypnotics and sedatives. Conversely, prepandemic rates of antidepressant prescriptions were already increasing, especially in adolescents, and this trend increase accelerated in the years following the pandemic.

We observed only moderate increases in prescription rates and trends for antipsychotics, as has been reported in previous studies.^10,37^ Antipsychotic medications are usually prescribed for the treatment of psychotic and conduct disorders and only as second-line treatments for major behavioral and bipolar disorders.^38,39^ Finally, despite a more steeply increasing trend after the pandemic onset, prescriptions of psychostimulants did not rise above expected rates in France, as opposed to other countries.^32,33,36^ This finding should be interpreted with caution, as the acceleration of the prescription trend may also be associated with a modification of methylphenidate prescribing conditions in France. Indeed, initiation of methylphenidate treatment was reserved for hospital pediatric specialists only until September 2021, when it was extended to include pediatric specialists in private practice.^40^ Despite this, psychostimulant prescription rates have not yet exceeded the expected rates because of a significant decrease—the largest among all of the psychotropic medication classes—in prescriptions in the initial pandemic period. Hence, prescribing of psychostimulant medications should continue to be monitored in the coming years.

It is unlikely that the observed increases in psychotropic medication prescribing were the consequence of increased screening and management of mood and anxiety disorders in children and adolescents after the pandemic, as availability of mental health services did not increase despite the development of telemedicine.^41^ Likewise, during the study period, there were no new psychotropic medications with a pediatric marketing authorization in France. Therefore, our findings likely reflect the actual mental health needs of children and adolescents after the onset of the pandemic, whether pre-existing or newly emerged. Indeed, the direct physical impact of the COVID-19 pandemic in children was low, but the indirect societal burden of the pandemic was considerable.^24,42^ Social isolation and school closures have been cited as main triggers of youth distress^23^; in France, however, school closures were mandated only in the first few months of the pandemic. Undeniably, the pandemic was associated with boosted social media growth and online peer interactions.^43,44^ The pandemic was also associated with an important economic fallout and a rise in domestic violence.^45^ The negative association of these stressors in adolescents, who already experience important physiological and psychological turmoil, is obvious but should not be neglected for younger children.^43,46^ In our study, increases in anxiolytic, hypnotic and sedative, and antidepressant prescriptions were also important in this population and underline the vulnerability of young children to stressful environments, with potential major repercussions on their future psychological trajectories.

### Strengths and Limitations

Our study has important strengths. First, we used a retail pharmacy dispensing database that readily extrapolates data at a national level. Furthermore, given that the rate of pediatric psychotropic medication prescriptions is known to be increasing in recent years,^47,48,49^ our findings were based on a time-series statistical analysis accounting for these prepandemic rates and trends.

This study has some limitations. First, previous studies have shown significant differences between men and women in the association between the pandemic and mental health and psychotropic medication use.^36,37,50^ The X-ponent database did not reliably record this information throughout the entire study period, preventing us from stratifying our analysis by sex. Interpretation of our study findings was also limited by the absence of information on medical diagnoses, which are not available in the X-ponent database.

## Conclusions

This cross-sectional study using time-series analysis found that prescribing of psychotropic medication for children and adolescents in France significantly and persistently increased after the onset of the COVID-19 pandemic. Prescription trends increased substantially for anxiolytic, hypnotic and sedative, and antidepressant medications that are generally prescribed to treat anxiety and mood disorders. Prescription rates rose above the expected rates for all psychotropic medication classes except psychostimulants. Changes were more pronounced in adolescents but were also significant in children. Future research should focus on the underlying determinants to adequately tailor medical treatment and public health and policy approaches to improve the psychological trajectories of children and adolescents.

## Notes

Supplement 1. **eMethods.** Calculation of rate ratios **eTable 1.** List of agents prescribed in metropolitan France for children and adolescents between January 2016 and May 2022. **eTable 2.** Changes in trends and rates of psychotropic prescriptions **eTable 3.** Changes in trends and rates of psychotropic prescriptions: primary and sensitivity analysis **eFigure 1.** Changes in trends and rates of psychotropic prescriptions: children and adolescents **eFigure 2.** Changes in trends and rates of antipsychotic prescriptions: children and adolescents **eFigure 3.** Changes in trends and rates of anxiolytic prescriptions: children and adolescents **eFigure 4.** Changes in trends and rates of hypnotic and sedative prescriptions: children and adolescents **eFigure 5.** Changes in trends and rates of antidepressant prescriptions: children and adolescents **eFigure 6.** Changes in trends and rates of psychostimulant prescriptions: children and adolescents **eFigure 7.** Correlograms and residual analysis of primary and sensitivity analyses

Supplement 2.
Data Sharing Statement
