# Supplementary material for: Psychotropic Medication Prescribing for Children and Adolescents After the Onset of the COVID-19 Pandemic
Source: JAMA Netw Open. 2024 Apr 23;7(4):e247965. doi: 10.1001/jamanetworkopen.2024.7965 (PMC11040414; doi:10.1001/jamanetworkopen.2024.7965)
Supplement: Supplement 1. — eMethods. Calculation of rate ratios eTable 1. List of agents prescribed in metropolitan France for children and adolescents between January 2016 and May 2022. eTable 2. Changes in trends and rates of psychotropic prescriptions eTable 3. Changes in trends and rates of psychotropic prescriptions: primary and sensitivity analysis eFigure 1. Changes in trends and rates of psychotropic prescriptions: children and adolescents eFigure 2. Changes in trends and rates of antipsychotic prescriptions: children and adolescents eFigure 3. Changes in trends and rates of anxiolytic prescriptions: children and adolescents eFigure 4. Changes in trends and rates of hypnotic and sedative prescriptions: children and adolescents eFigure 5. Changes in trends and rates of antidepressant prescriptions: children and adolescents eFigure 6. Changes in trends and rates of psychostimulant prescriptions: children and adolescents eFigure 7. Correlograms and residual analysis of primary and sensitivity analyses [file jamanetwopen-e247965-s001.pdf]

Supplementary Online Content

Valtuille Z, Aquaviva E, Trebossen V, et al. Psychotropic medication prescribing for children and adolescents after the COVID-19 pandemic. *JAMA Netw Open*. 2024;7(4):e247965. doi:10.1001/jamanetworkopen.2024.7965

eMethods. Calculation of rate ratios

eTable 1. List of agents prescribed in metropolitan France for children and adolescents between January 2016 and May 2022.

eTable 2. Changes in trends and rates of psychotropic prescriptions

eTable 3. Changes in trends and rates of psychotropic prescriptions: primary and sensitivity analysis

eFigure 1. Changes in trends and rates of psychotropic prescriptions: children and adolescents

eFigure 2. Changes in trends and rates of antipsychotic prescriptions: children and adolescents

eFigure 3. Changes in trends and rates of anxiolytic prescriptions: children and adolescents

eFigure 4. Changes in trends and rates of hypnotic and sedative prescriptions: children and adolescents

eFigure 5. Changes in trends and rates of antidepressant prescriptions: children and adolescents

eFigure 6. Changes in trends and rates of psychostimulant prescriptions: children and adolescents

eFigure 7. Correlograms and residual analysis of primary and sensitivity analyses

This supplementary material has been provided by the authors to give readers additional information about their work.

© 2024 Valtuille Z et al. *JAMA Network Open*.

eMethods. Calculation of rate ratios

The rate ratios were calculated at every point of time t (calendar months) of the period following the pandemic onset (June 1, 2020 to May 31, 2022) as the ratio between the monthly prescription rate estimated by the regression model and the expected monthly prescription rate forecast by the regression model but based only on pre-pandemic trends (counterfactual):

$$RR_t = \frac{\text{Monthly prescription rate estimated at time } t}{\text{Monthly prescription rate expected at time } t}$$

Confidence intervals were calculated using bootstrap. The rate ratios were cumulated and divided by the number of months, giving an average estimation for the entire period.

© 2024 Valtuille Z et al. *JAMA Network Open*.

eTable 1. **List of agents prescribed in metropolitan France for children and adolescents between January 2016 and May 2022**

Medication class	Agents
**Antipsychotics**	Amisulpride, Aripiprazole, Chlorpromazine, Clozapine, Cyamemazine, Flupentixol, Fluphenazine, Haloperidol, Levomepromazine, Lithium, Loxapine, Olanzapine, Paliperidone, Periciazine, Pimozide, Pipamperone, Pipotiazine, Quetiapine, Risperidone, Sulpiride, Tiapride, Zuclopenthixol
**Anxiolytics**	Alprazolam, Bromazepam, Buspirone, Captodiame, Clobazam, Clorazepate Potassique, Clotiazepam, Diazepam, Ethyle Loflazepate, Etifoxine, Hydroxyzine, Lorazepam, Nordazepam, Oxazepam, Prazepam
**Hypnotics and sedatives**	Estazolam, Loprazolam, Lormetazepam, Niaprazine, Nitrazepam, Zolpidem, Zopiclone
**Antidepressants**	Agomelatine, Amitriptyline, Amoxapine, Citalopram, Clomipramine, Dosulepine, Doxepine, Duloxetine, Escitalopram, Fluoxetine, Fluvoxamine, Imipramine, Maprotiline, Mianserine, Milnacipran, Mirtazapine, Moclobemide, Oxitriptan, Paroxetine, Sertraline, Tianeptine, Trimipramine, Venlafaxine, Vortioxetine
**Psychostimulants**	Deanol, Methylphenidate, Modafinil, Piracetam

eTable 2. Changes in rates and trends rates of psychotropic prescriptions

This table comprises the coefficients as calculated by the quasi-Poisson regression.

Outcome	Before the pandemic onset (Jan 2016 – Feb 2020)			Initial pandemic period (March – May 2020)		After the pandemic onset (June 2020 – May 2022)					
	**Level Jan. 2016[a] (95%CI)**	**Trend (95%CI)**	***p-value***	**Change in level (95%CI)**	***p-value***	**Change in level (95%CI)**	***p-value***	**Change in trend (95%CI)**	***p-value***	**Trend (95%CI)**	**May 2022 [a] (95 %CI)**
**Monthly prescription rate of psychotropic medications**	9.908 (9.620 to 10.204)	1.004 (1.003 to 1.004)	*<0.001*	0.885 (0.823 to 0.951)	*<0.001*	0.983 (0.949 to 1.018)	*0.34*	1.010 (1.008 to 1.012)	*<0.001*	1.013 (1.012 to 1.015)	16.1 (15.7 to 16.6)
Children	6.208 (5.997 to 6.424)	1.005 (1.004 to 1.005)	*<0.001*	0.883 (0.839 to 0.929)	*<0.001*	0.970 (0.935 to 1.007)	*0.12*	1.006 (1.004 to 1.009)	*<0.001*	1.011 (1.009 to 1.013)	10.5 (10.2 to 10.8)
Adolescents	13.635 (13.220 to 14.063)	1.003 (1.002 to 1.004)	*<0.001*	0.885 (0.819 to 0.956)	*0.002*	0.988 (0.952 to 1.025)	*0.51*	1.011 (1.009 to 1.013)	*<0.001*	1.014 (1.013 to 1.016)	21.6 (21.0 to 22.3)
**Monthly prescription rate of antipsychotics**	2.596 (2.526 to 2.668)	1.003 (1.002 to 1.004)	*<0.001*	0.984 (0.910 to 1.064)	*0.69*	1.011 (0.978 to 1.045)	*0.52*	1.007 (1.006 to 1.009)	*<0.001*	1.010 (1.009 to1.012)	4.1 (4.0 to 4.3)
Children	1.189 (1.156 to 1.223)	1.004 (1.003 to 1.005)	*<0.001*	0.980 (0.901 to 1.065)	*0.63*	1.014 (0.985 to 1.044)	*0.36*	1.003 (1.002 to 1.005)	*<0.001*	1.007 (1.006 to 1.009)	1.9 (1.9 to 2.0)
Adolescents	4.011 (3.901 to 4.125)	1.003 (1.002 to 1.004)	*<0.001*	0.984 (0.911 to 1.062)	*0.67*	1.007 (0.973 to 1.043)	*0.69*	1.008 (1.007 to 1.010)	*<0.001*	1.011 (1.010 to 1.013)	6.3 (6.1 to 6.5)
**Monthly prescription rate of anxiolytics**	2.715 (2.620 to 2.814)	0.998 (0.997 to 0.999)	*<0.001*	0.864 (0.823 to 0.907)	*<0.001*	1.084 (1.039 to 1.130)	*<0.001*	1.011 (1.009 to 1.014)	*<0.001*	1.009 (1.007 to 1.011)	3.2 (3.1 to 3.3)
Children	1.497 (1.451 to 1.544)	0.999 (0.998 to 1.000)	*0.03*	0.878 (0.845 to 0.913)	*<0.001*	1.051 (1.012 to 1.092)	*0.01*	1.004 (1.002 to 1.006)	*<0.001*	1.003 (1.001 to 1.004)	1.5 (1.4 to 1.5)
Adolescents	3.940 (3.791 to 4.096)	0.998 (0.997 to 0.999)	*<0.001*	0.856 (0.804 to 0.911)	*<0.001*	1.093 (1.044 to 1.146)	*<0.001*	1.014 (1.011 to 1.016)	*<0.001*	1.011 (1.009 to 1.014)	4.8 (4.7 to 5.0)
**Monthly prescription rate of hypnotics and sedatives**	0.207 (0.188 to 0.228)	0.972 (0.969 to 0.975)	*<0.001*	1.409 (1.228 to 1.616)	*<0.001*	1.430 (1.249 to 1.637)	*<0.001*	1.039 (1.034 to 1.044)	*<0.001*	1.010 (1.007 to 1.014)	0.1 (0.1 to 0.1)

© 2024 Valtuille Z et al. *JAMA Network Open*.

Children	0.033 (0.029 to 0.038)	0.985 (0.982 to 0.988)	*<0.001*	1.121 (0.879 to 1.415)	*0.35*	0.998 (0.830 to 1.196)	*0.98*	1.026 (1.015 to 1.038)	*<0.001*	1.010 (1.000 to 1.021)	0.0 (0.0 to 0.0)
Adolescents	0.380 (0.343 to 0.421)	0.971 (0.968 to 0.974)	*<0.001*	1.436 (1.241 to 1.662)	*<0.001*	1.477 (1.278 to 1.707)	*<0.001*	1.040 (1.035 to 1.045)	*<0.001*	1.010 (1.006 to 1.013)	0.2 (0.1 to 0.2)
**Monthly prescription rate of antidepressants**	1.120 (1.064 to 1.180)	1.005 (1.004 to 1.007)	*<0.001*	0.982 (0.902 to 1.069)	*0.67*	1.033 (0.980 to 1.090)	*0.23*	1.022 (1.020 to 1.025)	*<0.001*	1.028 (1.026 to 1.030)	3.3 (3.2 to 3.4)
Children	0.264 (0.253 to 0.275)	1.001 (0.999 to 1.002)	*0.42*	1.028 (0.976 to 1.083)	*0.30*	1.120 (1.062 to 1.180)	*<0.001*	1.007 (1.004 to 1.010)	*<0.001*	1.008 (1.005 to 1.011)	0.4 (0.4 to 0.4)
Adolescents	1.986 (1.884 to 2.093)	1.006 (1.004 to 1.007)	*<0.001*	0.974 (0.892 to 1.064)	*0.56*	1.022 (0.969 to 1.078)	*0.42*	1.023 (1.021 to 1.026)	*<0.001*	1.029 (1.027 to 1.031)	6.1 (5.9 to 6.3)
**Monthly prescription rate of psychostimulants**	4.062 (3.910 to 4.219)	1.007 (1.006 to 1.008)	*<0.001*	0.816 (0.770 to 0.863)	*<0.001*	0.915 (0.877 to 0.954)	*<0.001*	1.006 (1.004 to 1.009)	*<0.001*	1.014 (1.011 to 1.016)	7.3 (7.1 to 7.6)
Children	3.494 (3.358 to 3.635)	1.007 (1.006 to 1.008)	*<0.001*	0.851 (0.803 to 0.901)	*<0.001*	0.934 (0.896 to 0.974)	*0.002*	1.007 (1.004 to 1.009)	*<0.001*	1.014 (1.012 to 1.017)	7.1 (6.9 to 7.4)
Adolescents	4.630 (4.454 to 4.811)	1.007 (1.006 to 1.008)	*<0.001*	0.785 (0.740 to 0.833)	*<0.001*	0.896 (0.857 to 0.937)	*<0.001*	1.006 (1.003 to 1.009)	*<0.001*	1.013 (1.011 to 1.016)	7.5 (7.3 to 7.8)

[a] level corresponds to the monthly prescription rate per 1,000 children and adolescents estimated by the model
CI: confidence interval

© 2024 Valtuille Z et al. *JAMA Network Open*.

eTable 3. Changes in rates and trends of psychotropic prescriptions: primary and sensitivity analyses

Model	Before the onset of the pandemic (Jan 2016 – Feb 2020)			Initial pandemic period (March – May 2020)		After the onset of the pandemic (June 2020 – May 2022)					
	Level Jan. 2016[a] (95%CI)	Trend, % (95%CI)	*p-value*	Change in level, % (95%CI)	*p-value*	Change in level, % (95%CI)	*p-value*	Change in trend, % (95%CI)	*p-value*	Trend, % (95%CI)	Level May 2022[a] (95 %CI)
**Primary analysis**											
**Quasi-Poisson model**	9.9 (9.6 to 10.2)	0.4 (0.3 to 0.4)	*<0.001*	-11.5 (-17.7 to -4.9)	*<0.001*	-1.7 (-5.1 to 1.8)	*0.34*	1.0 (0.8 to 1.2)	*<0.001*	1.3 (1.2 to 1.5)	16.1 (15.7 to 16.6)
**Sensitivity analyses**											
**Quasi-Poisson model with Fourier terms**	9.6 (9.4 to 9.9)	0.4 (0.3 to 0.4)	*<0.001*	-10.8 (-21.0 to 0.8)	*0.07*	-0.6 (-4.9 to 3.9)	*0.79*	0.9 (0.7 to 1.2)	*<0.001*	1.3 (1.1 to 1.5)	15.2 (14.3 to 16.1)
**Negative binomial regression model**	10.0 (9.7 to 10.2)	0.4 (0.3 to 0.4)	*<0.001*	-11.5 (-17.6 to -5.1)	*<0.001*	-1.6 (-5.0 to 1.9)	*0.37*	1.0 (0.8 to 1.2)	*<0.001*	1.4 (1.2 to 1.5)	16.2 (15.7 to 16.7)

[a] level corresponds to the monthly prescription rate per 1,000 children and adolescents estimated by the model
CI: confidence interval

© 2024 Valtuille Z et al. *JAMA Network Open*.

eFigure 1. Changes in rates and trends of psychotropic prescriptions: children and adolescents

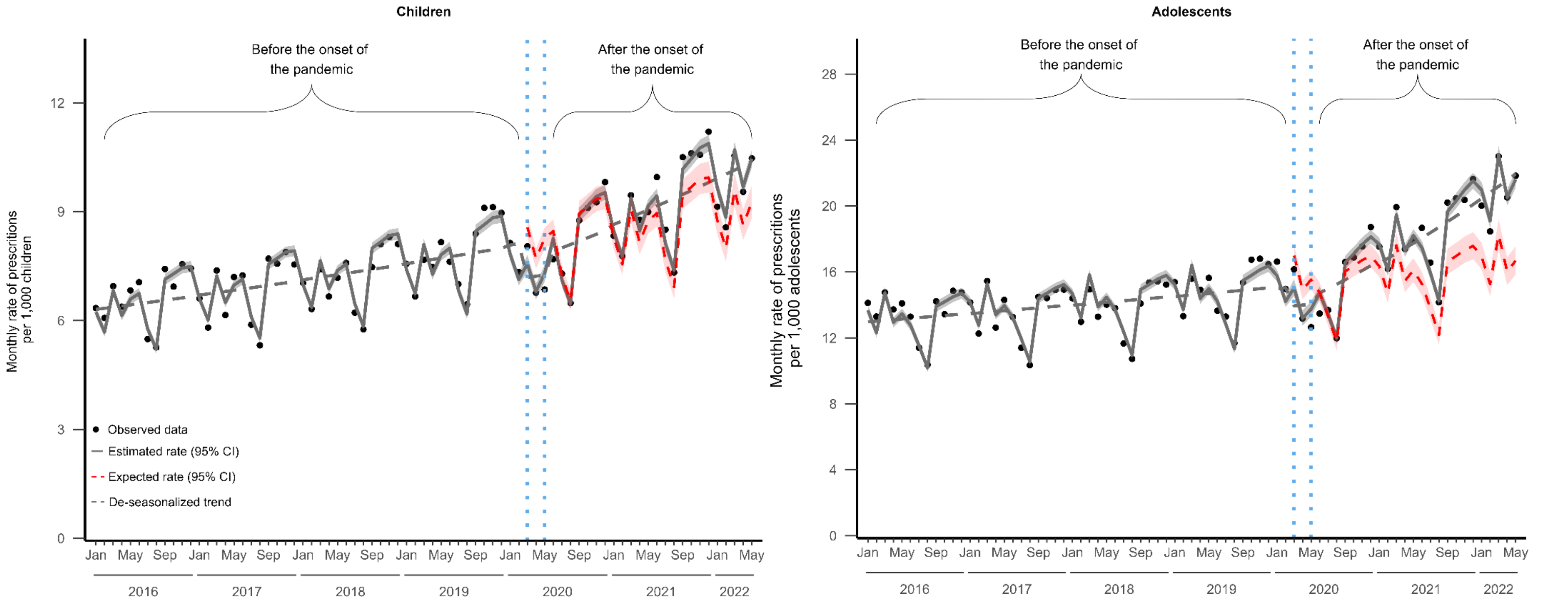

Black dots indicate observed data per 1,000 children (right) and adolescents (left). Grey bold lines indicate estimated prescription rates based on observed data using the quasi-Poisson regression model with corresponding 95% confidence intervals. Red dotted lines indicate the expected rates based on pre-pandemic observed data using the quasi-Poisson model. Grey dotted lines indicate estimated prescription trends using a de-seasonalized quasi-Poisson regression model. Vertical broken lines show the initial pandemic period between March and May 2020. Before the COVID-19 pandemic onset: January 2016 to February 2020. After the COVID-19 pandemic onset: June 2020 to May 2022.

95%CI: 95% confidence interval

© 2024 Valtuille Z et al. *JAMA Network Open*.

eFigure 2. Changes in trends and rates of antipsychotic prescriptions: children and adolescents

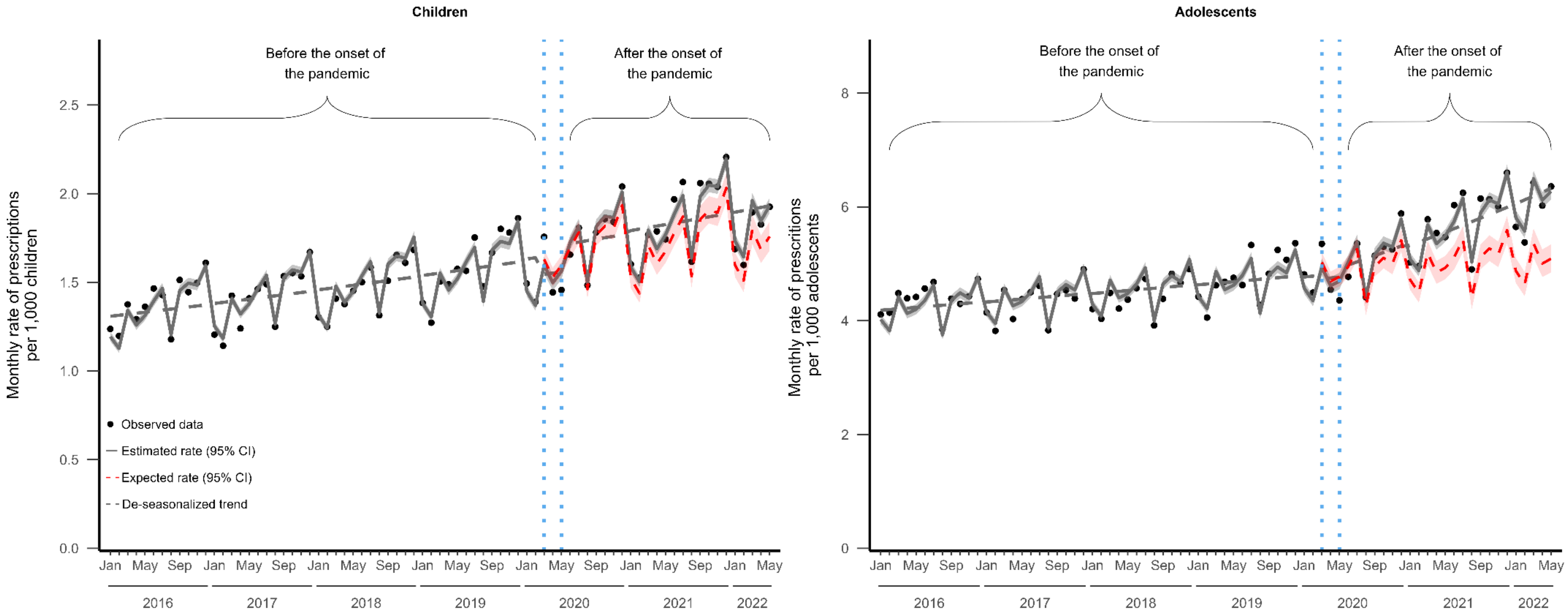

Black dots indicate observed data per 1,000 children (right) and adolescents (left). Grey bold lines indicate estimated prescription rates based on observed data using the quasi-Poisson regression model with corresponding 95% confidence intervals. Red dotted lines indicate the expected rates based on pre-pandemic observed data using the quasi-Poisson model. Grey dotted lines indicate estimated prescription trends using a de-seasonalized quasi-Poisson regression model. Vertical broken lines show the initial pandemic period between March and May 2020. Before the COVID-19 pandemic onset: January 2016 to February 2020. After the COVID-19 pandemic onset: June 2020 to May 2022.

95%CI: 95% confidence interval

© 2024 Valtuille Z et al. *JAMA Network Open*.

eFigure 3. Changes in trends and rates of anxiolytic prescriptions: children and adolescents

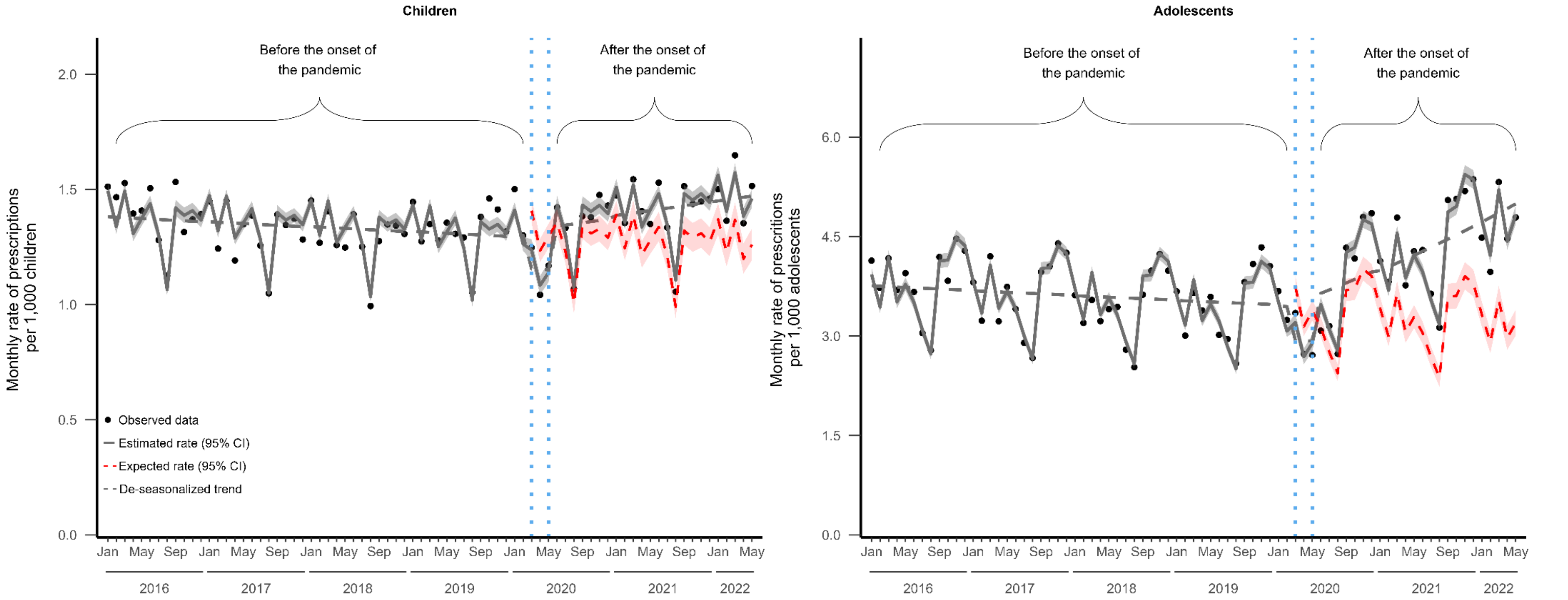

Black dots indicate observed data per 1,000 children (right) and adolescents (left). Grey bold lines indicate estimated prescription rates based on observed data using the quasi-Poisson regression model with corresponding 95% confidence intervals. Red dotted lines indicate the expected rates based on pre-pandemic observed data using the quasi-Poisson model. Grey dotted lines indicate estimated prescription trends using a de-seasonalized quasi-Poisson regression model. Vertical broken lines show the initial pandemic period between March and May 2020. Before the COVID-19 pandemic onset: January 2016 to February 2020. After the COVID-19 pandemic onset: June 2020 to May 2022.

95%CI: 95% confidence interval

© 2024 Valtuille Z et al. *JAMA Network Open*.

eFigure 4. Changes in trends and rates of hypnotics and sedatives prescriptions: children and adolescents

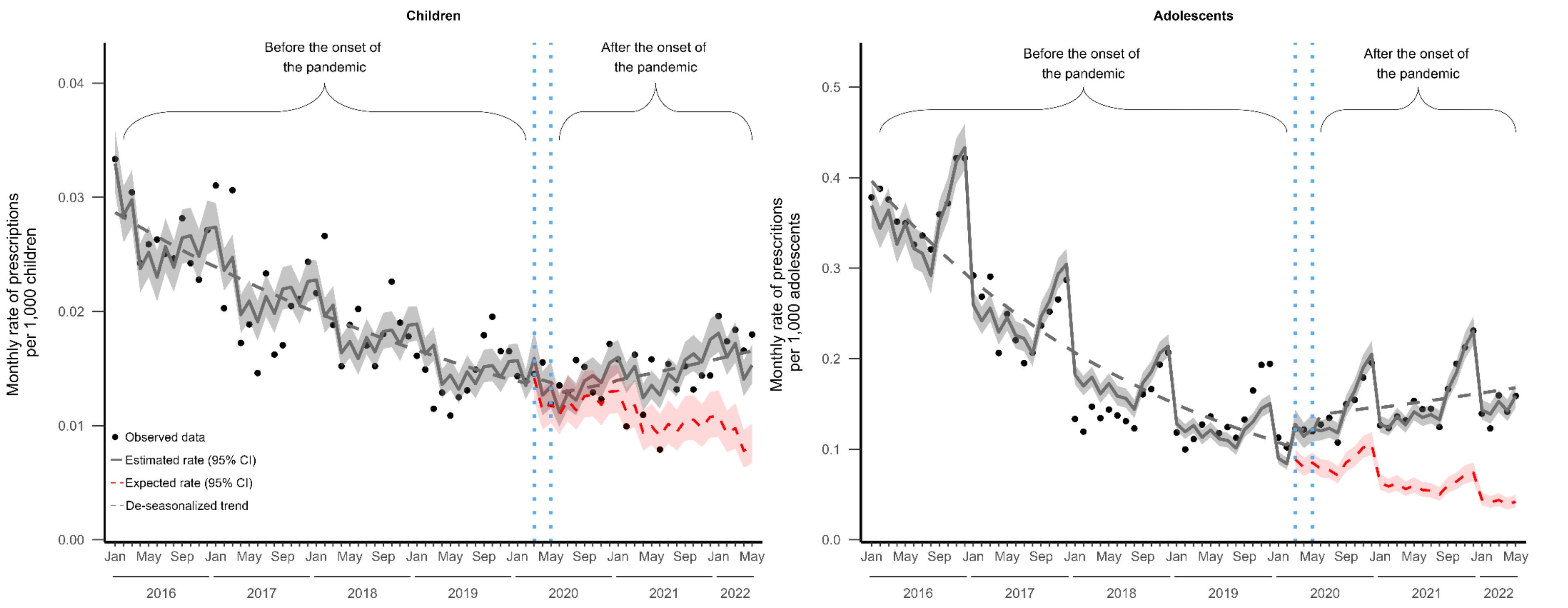

Black dots indicate observed data per 1,000 children (right) and adolescents (left). Grey bold lines indicate estimated prescription rates based on observed data using the quasi-Poisson regression model with corresponding 95% confidence intervals. Red dotted lines indicate the expected rates based on pre-pandemic observed data using the quasi-Poisson model. Grey dotted lines indicate estimated prescription trends using a de-seasonalized quasi-Poisson regression model. Vertical broken lines show the initial pandemic period between March and May 2020. Before the COVID-19 pandemic onset: January 2016 to February 2020. After the COVID-19 pandemic onset: June 2020 to May 2022.

95%CI: 95% confidence interval

© 2024 Valtuille Z et al. *JAMA Network Open*.

eFigure 5. Changes in trends and rates of antidepressant prescriptions: children and adolescents

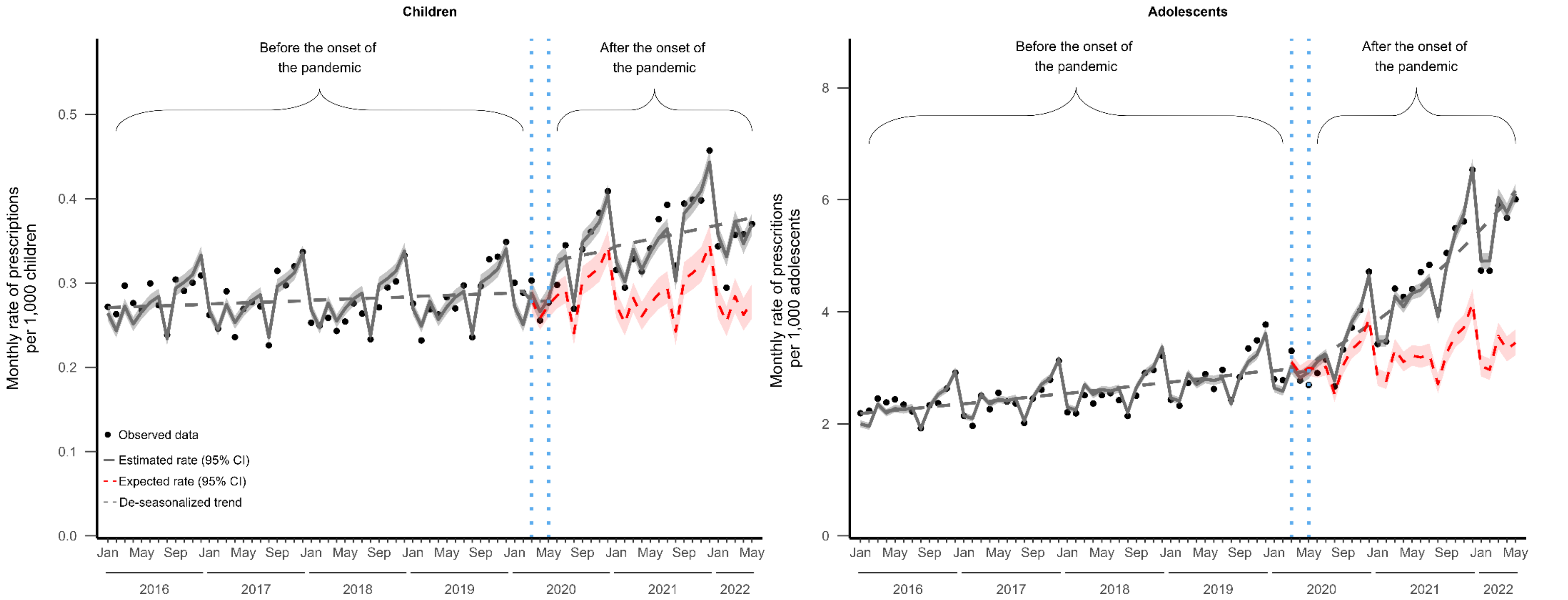

Black dots indicate observed data per 1,000 children (right) and adolescents (left). Grey bold lines indicate estimated prescription rates based on observed data using the quasi-Poisson regression model with corresponding 95% confidence intervals. Red dotted lines indicate the expected rates based on pre-pandemic observed data using the quasi-Poisson model. Grey dotted lines indicate estimated prescription trends using a de-seasonalized quasi-Poisson regression model. Vertical broken lines show the initial pandemic period between March and May 2020. Before the COVID-19 pandemic onset: January 2016 to February 2020. After the COVID-19 pandemic onset: June 2020 to May 2022.

95%CI: 95% confidence interval

© 2024 Valtuille Z et al. *JAMA Network Open*.

eFigure 6. Changes in trends and rates of psychostimulant prescriptions: children and adolescents

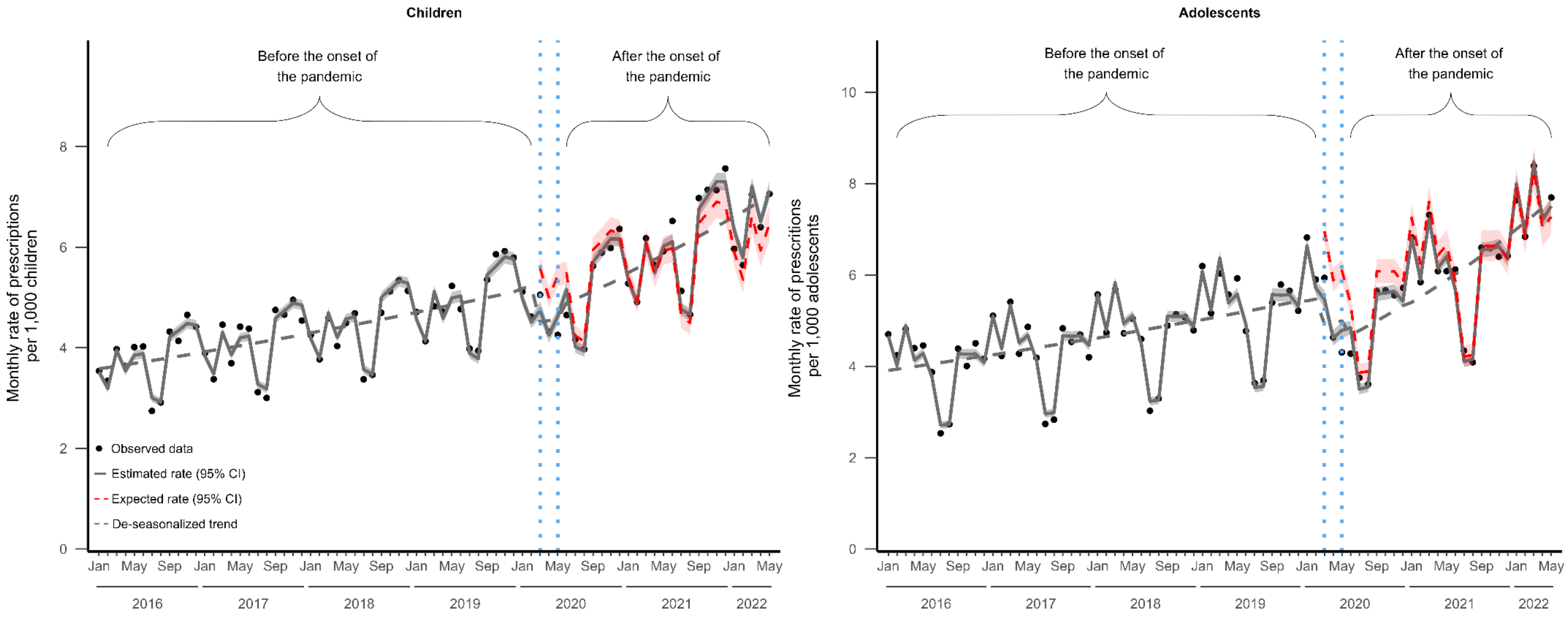

Black dots indicate observed data per 1,000 children (right) and adolescents (left). Grey bold lines indicate estimated prescription rates based on observed data using the quasi-Poisson regression model with corresponding 95% confidence intervals. Red dotted lines indicate the expected rates based on pre-pandemic observed data using the quasi-Poisson model. Grey dotted lines indicate estimated prescription trends using a de-seasonalized quasi-Poisson regression model. Vertical broken lines show the initial pandemic period between March and May 2020. Before the COVID-19 pandemic onset: January 2016 to February 2020. After the COVID-19 pandemic onset: June 2020 to May 2022.

95%CI: 95% confidence interval

© 2024 Valtuille Z et al. *JAMA Network Open*.

eFigure 7. Correlograms and residual analysis of primary and sensitivity analyses

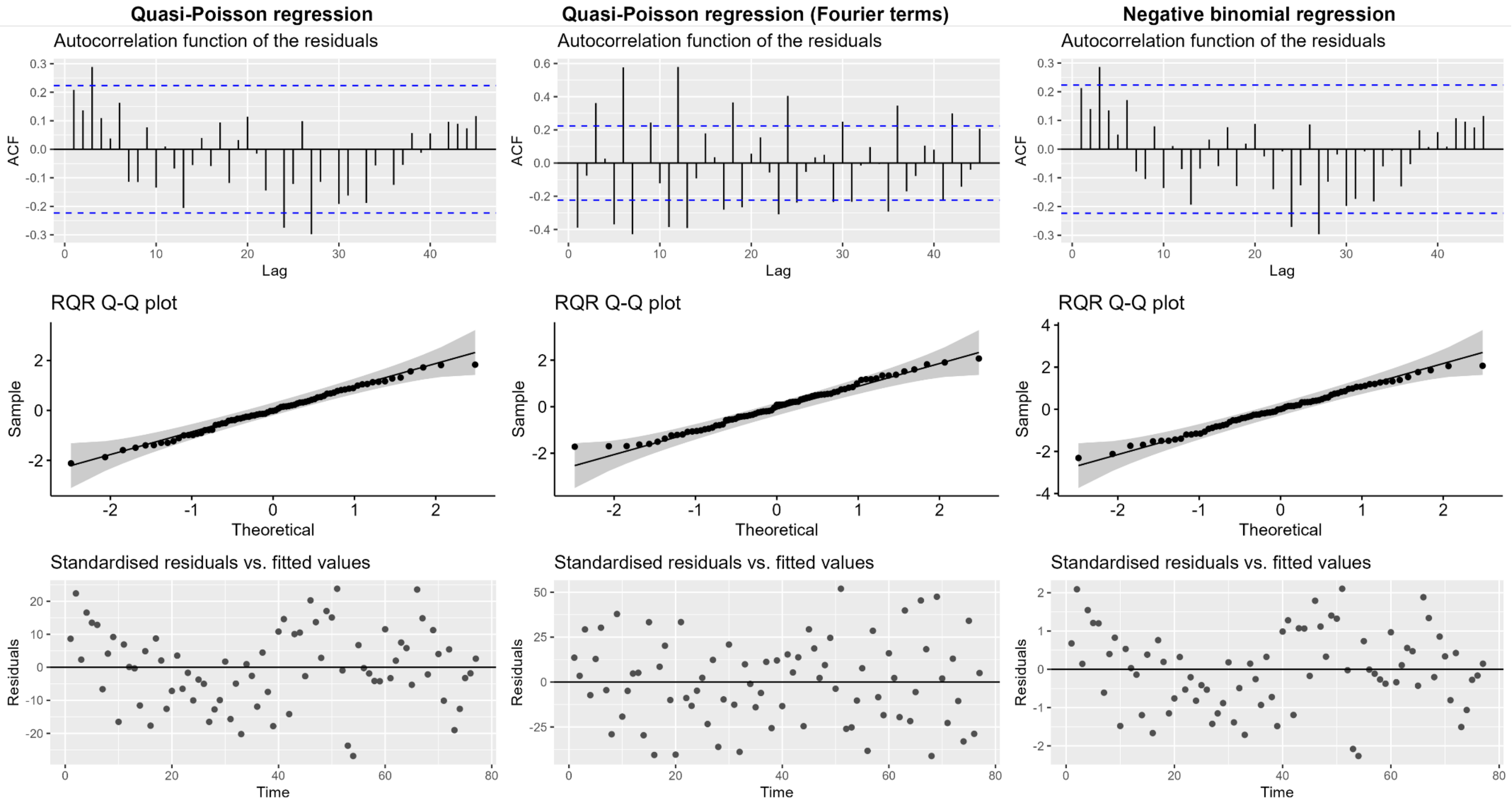

RQR: Randomized Quantile Residuals.

© 2024 Valtuille Z et al. *JAMA Network Open*.
