# Supplementary material for: Psychotropic Medication Prescribing for Children and Adolescents After the Onset of the COVID-19 Pandemic
Source: JAMA Netw Open. 2024 Apr 23;7(4):e247965. doi: 10.1001/jamanetworkopen.2024.7965 (PMC11040414; doi:10.1001/jamanetworkopen.2024.7965)
Supplement: Supplement 2. — Data Sharing Statement [file jamanetwopen-e247965-s002.pdf]

Data Sharing Statement

Data
Data available: No

Additional Information
Explanation for why data not available: The clinical codes, data management and analysis code used in this study are available on request from the corresponding author. Access to the data dictionary and these anonymized data can be granted upon request to IQVIA France. FK and ZV both had full access to all the data in the study and take responsibility for the integrity of the data and the accuracy of the data analysis.
